# Elevated Toll-Like Receptor-Induced CXCL8 Secretion in Human Blood Basophils from Allergic Donors Is Independent of Toll-Like Receptor Expression Levels

**DOI:** 10.1371/journal.pone.0149275

**Published:** 2016-02-12

**Authors:** Markus Steiner, Thomas Hawranek, Michael Schneider, Fatima Ferreira, Jutta Horejs-Hoeck, Andrea Harrer, Martin Himly

**Affiliations:** 1 Department of Molecular Biology, University of Salzburg, Salzburg, Austria; 2 Laboratory for Immunological and Molecular Cancer Research, Paracelsus Medical University, Salzburg, Austria; 3 Department of Dermatology, Paracelsus Medical University, Salzburg, Austria; 4 Bühlmann Laboratories AG, Schönenbuch, Switzerland; 5 Department of Neurology, Paracelsus Medical University, Salzburg, Austria; University of Bergen, NORWAY

## Abstract

Human blood basophils have recently gained interest in addition to their function as allergic effector cells. Previous work suggests the involvement of innate immune mechanisms in the development and exacerbation of allergic responses, which might be mediated by basophils. We assayed the expression levels of Toll-like receptor (TLR) 1, 2, 4 and 6 on purified basophils from birch pollen-, house dust mite-, and non-allergic individuals. Additionally, we compared cytokine and chemokine secretion upon TLR stimulation in these basophil donor groups. Expression of TLR4 on the basophils of the allergic donor groups was decreased and CXCL8 secretion was elevated upon stimulation of TLR1/2 and TLR2/6 compared to the non-allergic donors. Decreased TLR expression and elevated CXCL8 secretion may represent possible mechanisms for aggravation of allergic symptoms in case of parasitic infection.

## Introduction

TLRs are central molecules of innate immune responses, as they interact with pathogen-associated molecular patterns derived from microbial invaders[1]. Recent publications suggest that TLRs may not only be involved in host defense against microbial infection, but may also contribute to the formation and exacerbation of allergic responses by influencing IgE-mediated pathways. Tulic *et al*.[2] compared TLR function and expression in allergic versus non-allergic children. They found excessive TLR function in allergic children at birth and a relative decrease with age thereafter, which they discussed to result in an upregulated T_H_2 response. A link between TLR signaling and allergy was further strengthened by the studies of Kucuksezer *et al*.[3] who showed that stimulation of myeloid dendritic cells via TLR4 or TLR8 resulted in enhanced allergen-specific CD4^+^ T cell proliferation[3].

Human basophils are, although in very low abundance in blood, important key players in allergic reactions as they release first line defense mediators and T_H_2 skewing cytokines upon allergen-mediated crosslinking of Fc epsilon receptor 1 (FcεRI)[4]. The presence of TLRs in basophils was investigated by Sabroe *et al*.[5] detecting mRNA of TLR2 and 4 in human basophils. This study was corroborated by Komyia *et al*.[6] who found TLR2, 4, 6, and in some donors also TLR1 on a transcriptional level. Bieneman *et al*.[7] confirmed reactivity of human blood basophils to ligands of TLR2 but not to TLR4, whereas Watanabe *et al*.[8] showed IgG1 and IgG4 production by B cells mediated upon basophil activation by TLR2 and 4 ligands from patients with autoimmune pancreatitis. However, an involvement of human blood basophils via their TLRs in inflammatory or immunomodulatory processes like allergy is still scarce. Gyimesi *et al*.[9] showed a slight upregulation of one activation marker CD63 and histamine release in basophils from atopic patients at high concentrations of TLR4 ligand. Suurmond *et al*.[10] observed an increased cytokine release from basophils upon simultaneous stimulation of the high-affinity IgE receptor and TLRs. It was shown that TLR4 or TLR9 stimulation combined with FcεRI activation by anti-IgE resulted in a synergistic increase of IL-4, CXCL8, IL-13 and RANTES/CCL5, all of which are thought to enhance IgE-mediated responses, such as allergy or parasitic infection.

To further investigate the potential role of TLRs in augmenting allergic symptoms mediated by basophils, we compared TLR expression levels between allergic and non-allergic individuals and analyzed their capacity to trigger mediator release upon TLR stimulation of the different donor groups. We analyzed the surface expression of TLR1, 2, 4 and 6 on purified human basophils from non-allergic, birch pollen (BP)-allergic, and house dust mite (HDM)-allergic donors. In addition, basophils were stimulated with TLR ligands Pam_3_CSK_4_ (TLR1/2), Pam_2_CSK_4_ (TLR2/6) and LPS (TLR4) in order to investigate cyto-/chemokine release (CXCL8, IL-6, IFN-γ, TNF-α) and basophil activation marker CD203c surface expression.

## Materials and Methods

### Basophil donors

70 ml EDTA-anticoagulated whole blood were collected from birch pollen-allergic, house dust mite-allergic, and non-allergic donors (n = 8, each; Table 1). Next to clinical history, allergies were confirmed *in vivo* by positive skin prick tests (Pangramin, ALK-Abelló, Horsholm, Denmark). ImmunoCAP^™^ (Thermo Scientific, Waltham, MA, USA) was used to quantify allergen-specific IgE. IgE reactivity ≥ 0.7 kU/l was considered positive (Table 1). This study and its experiments were performed in accordance with the local institutional guidelines strictly adhering to methods and experimental protocols approved by the Ethics Committee of Land Salzburg (No.: 415-E/1/1117-2009). Written informed consents were obtained from the blood donors.

**Table 1:** Allergic patients and non-allergic donors.

Donor # (donor group)	Age	Sex	Symptoms	Symptoms to	SPT	RAST classes	Total IgE (kU/l)
1 (NA)	36	m	-	-	n.d.	n.d.	n.d.
2 (NA)	30	m	-	-	n.d.	n.d.	n.d.
3 (BP)	22	f	RC (March-August)	tree pollen, grass pollen	birch, hazel, grass, plantain, mugwort	3 birch, 3 Bet v 1, 4 timothy, 3 Phl p 5b, 2 plantain, 2 mugwort	107
4 (HDM)	30	f	RC (at night/in morning)	HDM	HDM	3 HDM, 3 Der p 1, 3 Der p 2, 4 D. farinae	65.9
5 (HDM)	29	f	RC, asthma	HDM, cat	HDM, L. destructor, tyrophagus, grass, cat	5 HDM, 3 Der p 1, 3 Der p 2, 3 L. destructor	349
6 (HDM)	14	m	RC	HDM, grass pollen	HDM, D. farinae, grass, plantain	3 HDM, 3 Der p 1, 3 timothy, 3 Phl p 1	41.9
7 (BP)	49	m	RC (February-May)	tree pollen, horse	birch, alder, hazel, horse	5 birch, 5 Bet v 1	309
8 (HDM)	56	f	RC (at night/in morning)	HDM	HDM	4 HDM, 3 Der p 1, 4 Der p 2	708
9 (BP)	13	m	RC (March-May)	tree pollen	birch, alder	5 birch, 5 Bet v 1, 1 timothy, 1 plantain	122
10 (BP)	43	f	RC (March-May)	tree pollen	birch, alder, hazel,	4 birch, 4 Bet v 1	59.9
11 (BP)	8	m	RC (March-July)	tree pollen, grass pollen	n.d.	4 birch, 5 timothy	456
12 (HDM)	31	m	RC (at night/in morning)	HDM	HDM, grass	3 HDM, 3 Der p 1, 3 Der p 2, 2 E. maynei, 3 timothy, 3 Phl p 1	142
13 (HDM)	26	f	RC (at night)	HDM	n.d.	2 Der p 1, 4 Der p 2, 3 Der f 1, 4 Der f 2, 3 L. destructor	n.d.
14 (HDM)	31	m	RC, asthma (at night)	HDM	HDM, L. destructor, tyrophagus, grass	5 HDM, 5 Der p 1, 4 Der p 2, 3 timothy, 3 Phl p 1	659
15 (BP)	43	f	RC (February-April)	tree pollen	birch, ash, alder, hazel, plantain	5 birch, 3 ash, 3 Ole e 1, 2 timothy	198
16 (BP)	42	f	RC (March-April)	tree pollen, grass pollen	birch, alder, grass, plantain, pigweed	2 birch, 2 Bet v 1, 3 timothy, 2 Phl p 1, 3 Phl p 5b	34.9
17 (BP)	30	f	RC (February-May)	tree pollen	birch, alder, hazel	3 birch, 3 Bet v 1, 3 ash	38.9
18 (NA)	49	w	-	-	n.d.	n.d.	n.d.
19 (NA)	53	m	-	-	n.d.	n.d.	n.d.
20 (NA)	28	w	-	-	n.d.	n.d.	n.d.
21 (NA)	32	w	-	-	n.d.	n.d.	n.d.
22 (HDM)	23	m	nasal obstruction, RC	HDM, grass pollen	HDM, grass, cat	2 HDM, 2 Der p 1, 2 Der p 2, 1 D. farinae, 1 timothy, 2 Phl p 1, 3 cat, 2 dog	49.6
23 (NA)	25	w	-	-	n.d.	n.d.	n.d.
24 (NA)	29	m	-	-	n.d.	n.d.	n.d.

SPT, skin prick test; NA, non-allergic; BP, birch pollen; HDM, house dust mite; RC; rhinoconjunctivitis; D. farinae, Dermatophagoides farinae; L. destructor, Lepidoglyphus destructor; E. maynei, Euroglyphus maynei; n.d., not determined

### Basophil purification

EDTA whole blood diluted 1:2 using DPBS (Sigma-Aldrich) was pipetted on top of 12 ml of a mixture (17:1) of Ficoll-Paque^™^ PLUS (GE Healthcare, Little Chalfont, UK) and Percoll^®^ (Sigma-Aldrich, St. Louis, MO, USA). Upon 30 minutes centrifugation at 500 g the PBMC-containing interphase was collected and washed. Basophils were purified by negative magnetic sorting using the Basophil Isolation Kit II (Miltenyi Biotec, Bergisch Gladbach, Germany). All buffers and solutions were without calcium and magnesium and pre-chilled at 4°C. Basophil purity was determined using flow cytometry by staining the purified cell fraction with anti-CCR3-PE antibody as well as an anti-CD203c-PE-Dy647 antibody (both from Bühlmann Laboratories, Schönenbuch, Switzerland). For purity determination, non-stimulated samples were compared to anti-FcεRI antibody (c = 375 ng/ml; Bühlmann Laboratories) stimulated samples and the CCR3 positive/CD203c shifting population gated.

### TLR staining and flow cytometry

The highly purified basophils of all donors were resuspended in stimulation buffer (IMDM, Sigma-Aldrich) supplemented with 10% heat-inactivated fetal bovine serum, 2 mM L-glutamine, 100 U/ml penicillin, 100 μg/ml streptomycin (all from Gibco^®^, Life Technologies, Carlsbad, CA, USA), and 2.5 ng/ml IL-3 (Sigma-Aldrich). 40.000 cells in 100 μl were used per reaction. Basophils were incubated for 30 minutes at 4°C with anti-TLR1-PE (clone GD2.F4), anti-TLR2-eFluor450 (clone T2.5), anti-TLR4-APC (clone HTA125), and anti-TLR6-Biotin (clone h-Per6) (20 minutes followed by 10 minutes Streptavidin-APC-eFluor780 treatment) or the respective isotype controls (eBioscience, San Diego, CA, USA and ImmunoTools, Friesoythe, Germany). Specificity of the anti-TLR antibodies has previously been demonstrated[11–14]. The cells were measured on a FACS Canto II (BD, Franklin Lakes, NJ, USA).

### Basophil stimulation and cytokine/chemokine determination

20,000 purified basophils in 200 μl per reaction were stimulated for 14 hours at 37°C and 7% CO_2_ in stimulation buffer (same composition as above) containing either TLR1/2 ligand Pam_3_CSK_4_ (c = 300 ng/ml), TLR2/6 ligand Pam_2_CSK_4_ (c = 100 ng/ml) (both from Invivogen, Toulouse, France), TLR4 ligand LPS (c = 5 μg/ml; Sigma-Aldrich). Secretion of IL-6, CXCL8 (IL-8), IFN-γ, and TNF-α was investigated from supernatant using FlowCytomix^™^ (eBioscience). A non-stimulated sample of basophils incubated in stimulation buffer only served as negative control. Anti-FcεRI antibody (c = 375 ng/ml) and fMLF (c = 1 μg/ml; both Bühlmann Laboratories) were used as positive controls for CD203c expression. Basophils were stained with anti-CD203c-PE-Dy647 antibody (Bühlmann Laboratories) to investigate CD203c upregulation by flow cytometry. The experiments were performed on 7 donors from each of the three groups.

### Statistics

Due to small sample size, analyses were performed using non-parametric tests. Wilcoxon matched pairs test was used to compare differences between isotype control and TLR antibody staining of both, allergic patients and non-allergic donors. Kruskal-Wallis test with Dunn’s *post hoc* test was used to compare (i) TLR expression of HDM-allergic patients, BP-allergic patients, and non-allergic donors, and (ii) CD203c upregulation of non-stimulated and stimulated (anti-FcεRI, fMLF, and the TLR ligands) basophils. Two-way repeated measures ANOVA with Bonferroni’s *post hoc* test was performed for multiple comparisons of cytokine and chemokine secretion from stimulated versus non-stimulated basophils of HDM- and BP-allergic patients and non-allergic donors. P<0.05 was considered statistically significant. Analyses were performed with Graph Pad Prism. (1992–2007 GraphPad Software, Inc., version 5)

## Results

**Fig 1:**
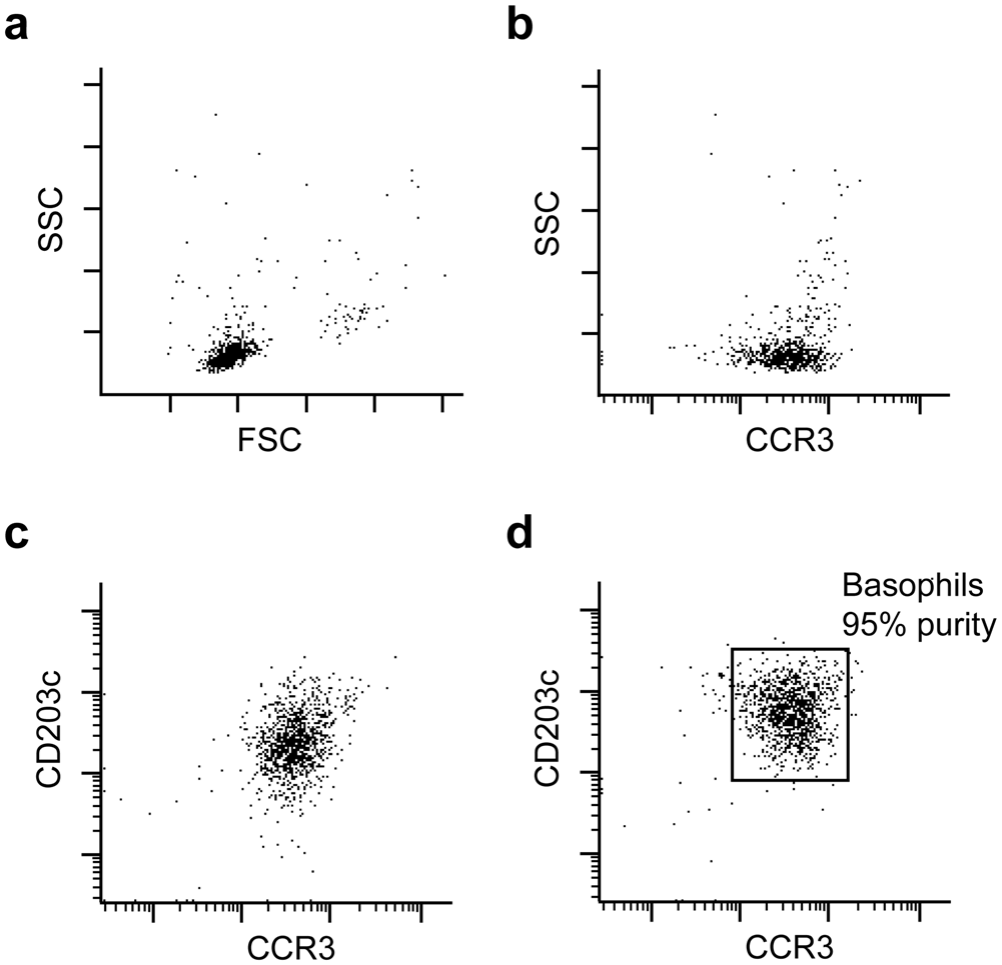
Determination of basophil purity. (**a**), Forward/side scatter plot of purified basophil fraction. (**b**), CCR3 expression of the recorded events. (**c**), a representative non-stimulated cell sample showing a putative basophil population. (**d**), upon stimulation with anti-FcεRI the CCR3 positive/CD203c shifting population was gated for purity determination. In this representative sample basophil purity was 95%.

This work was performed using highly purified basophils (>90%) from allergic and non-allergic individuals (Fig 1).

**Fig 2:**
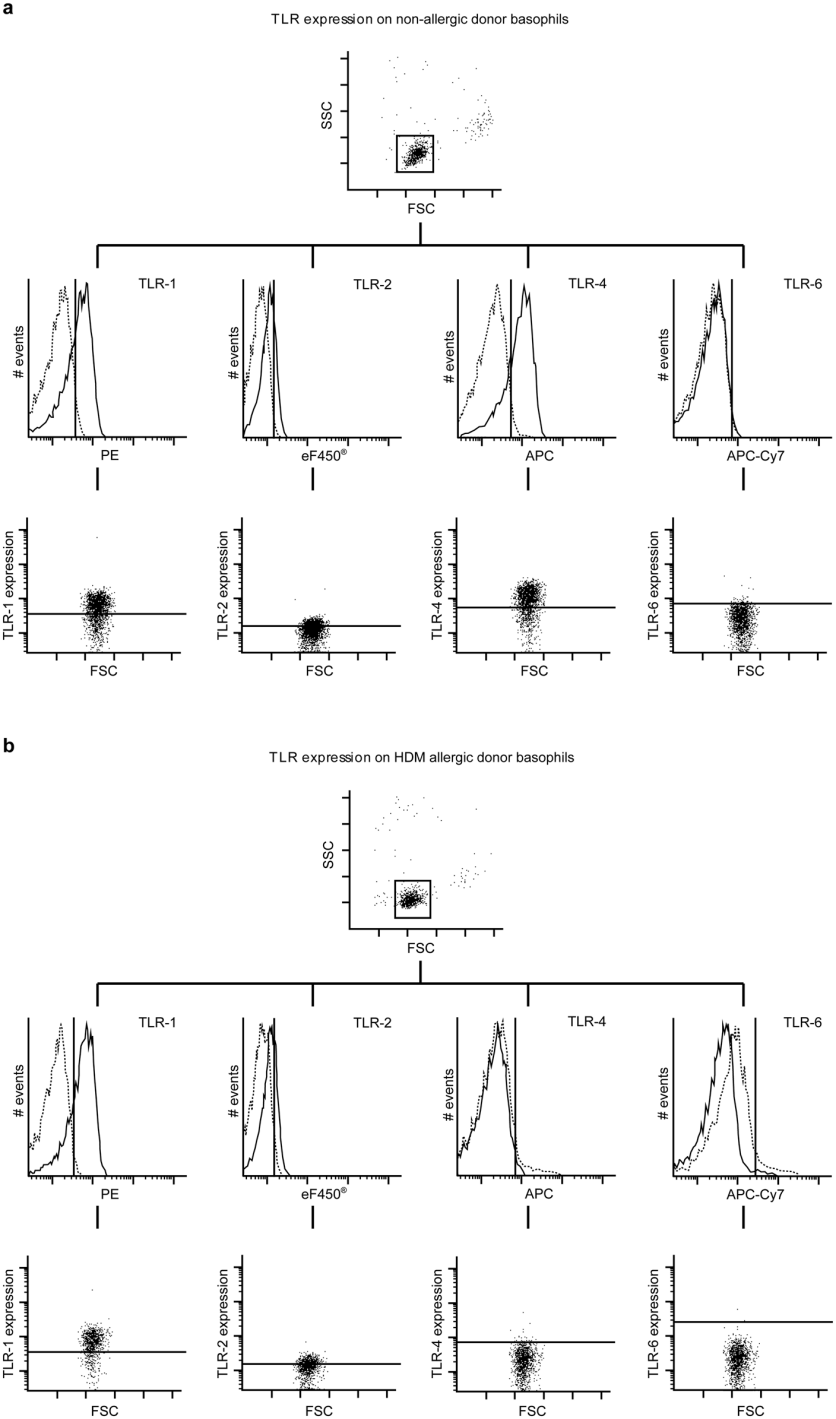
Gating strategy. Shown are one representative non-allergic donor (a) and one house dust mite (HDM)-allergic patient (b).

The gating strategy comparing TLR expression of allergic patients and non-allergic donors is depicted in Fig 2.

### TLR4 expression is altered in allergic basophil donors

**Fig 3:**
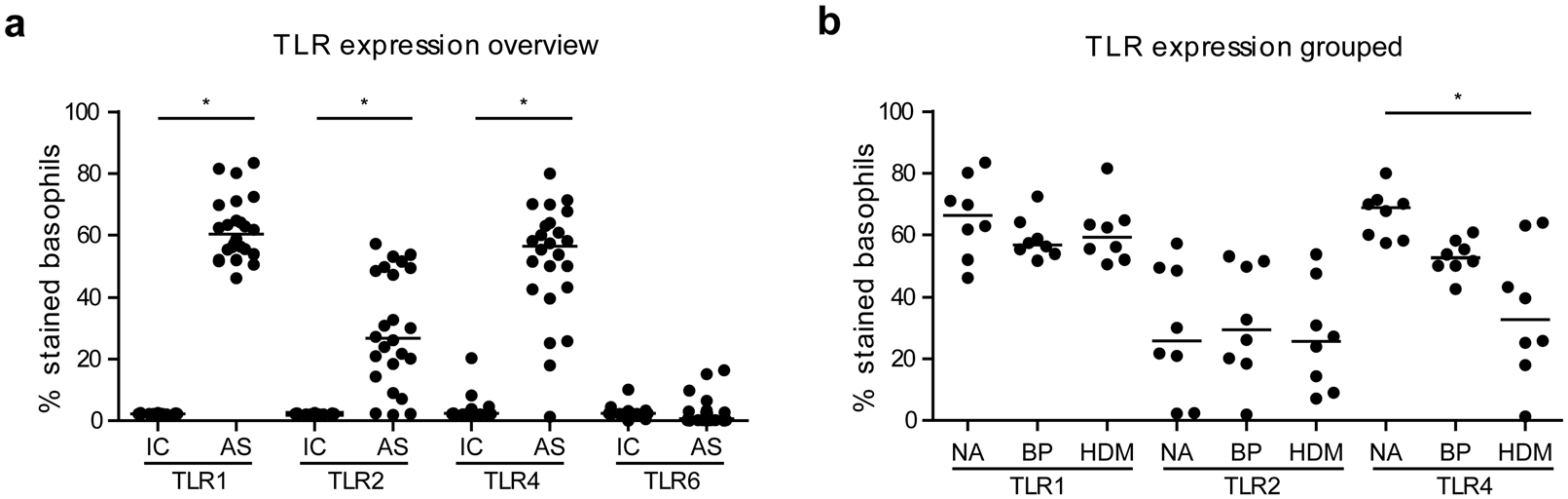
Overview of TLR expression on purified human basophils. Expression levels of significantly detectable TLR1, 2, and 4 were investigated between non-allergic (NA), birch pollen (BP)-allergic and house dust mite (HDM)-allergic donors (n = 8, each): (**a**), group-wise (n = 24 each) comparisons of TLR expressions (antibody staining, AS) with the respective isotype controls (IC); (**b**), comparison of TLR1, 2, 4 expression between non-allergic (NA) donors and HDM-/PB-allergic patients (n = 8, each). Group comparisons were performed by Wilcoxon matched pairs test in (a) and Kruskal-Wallis test with Dunn’s *post hoc* test in (b). Bars represent medians. NS, non-stimulated; *, p<0.05.

Expression of TLR1, TLR4 and to a lesser extent TLR2 was observed on up to 60% of basophils in all donor groups, whereas TLR6 expression was generally very low and could only be detected in 4 donors (Fig 3a and 3b). Comparative analysis of TLR1 and TLR2 revealed almost similar expression levels in non-allergic *vs*. allergic donor groups. TLR4 levels were significantly lower in HDM-allergic patients compared to non-allergic donors (Fig 3b).

### CXCL8 secretion from allergic donor basophils is augmented upon TLR1/2, TLR2/6 stimulation

**Fig 4:**
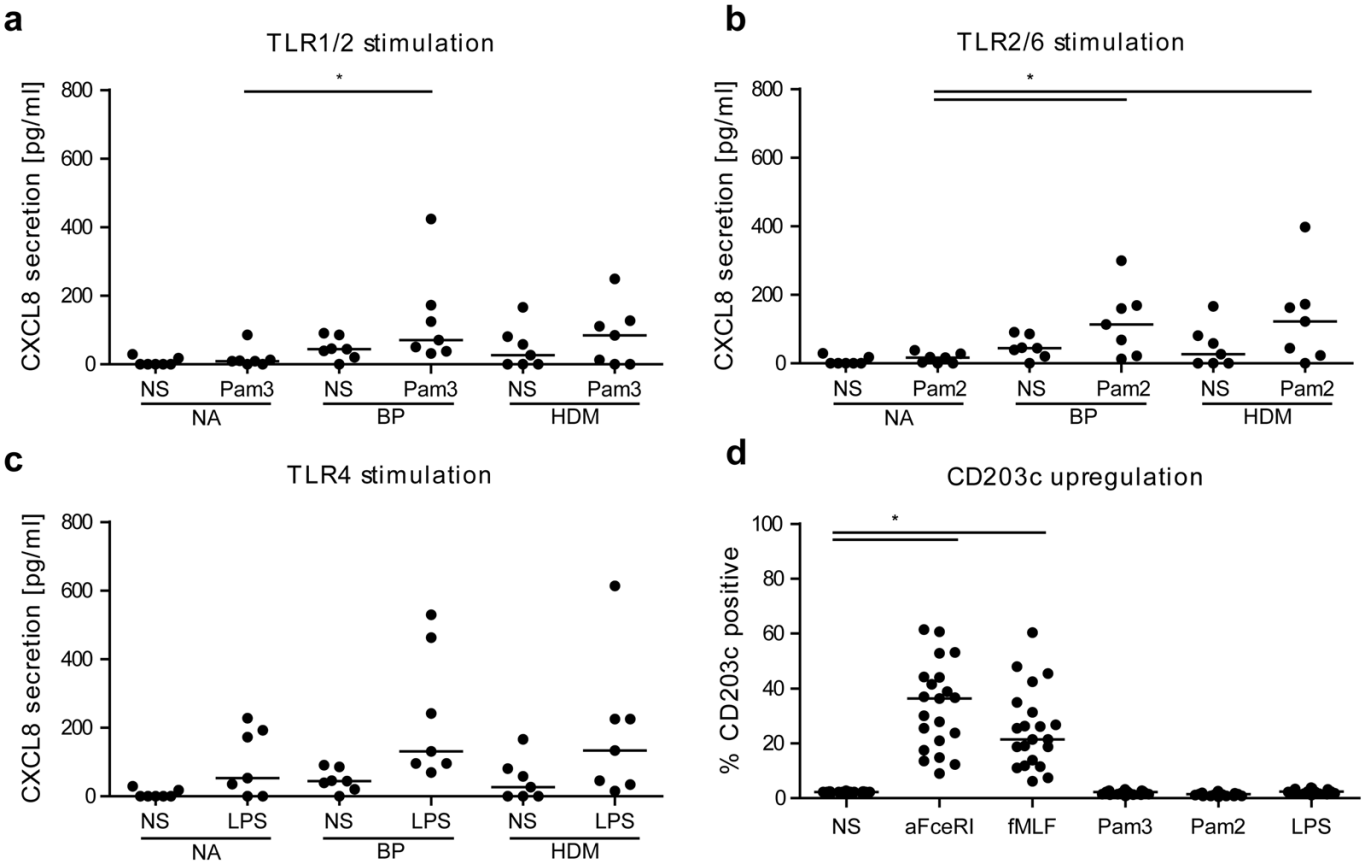
CXCL8 secretion and CD203c expression of purified human basophils of non-allergic (NA), birch pollen (BP)-allergic, and house dust mite (HDM)-allergic donors (n = 7, each). Stimulation of TLR1/2 with Pam_3_CSK_4_ (**a**), of TLR2/6 with Pam_2_CSK_4_ (**b**) and of TLR4 with LPS (**c**). (**d**), CD203c expression of all donors (n = 21); upon stimulation with positive controls (anti-FcεRI, fMLF) and the three TLR ligands (Pam_3_CSK_4_, Pam_2_CSK_4_, and LPS). Group comparisons were performed by two-way repeated measures ANOVA (a, b, c) with Bonferroni’s *post hoc* test and Kruskal-Wallis test with Dunn’s *post hoc* test (d). Bars represent medians. NS, non-stimulated; *, p<0.05.

From the cyto-/chemokines tested, we observed differences in release only for CXCL8. Stimulation with TLR1/2 ligand Pam_3_CSK_4_ (Fig 4a) resulted in upregulation of CXCL8 secretion in basophils from BP-allergic donors (p<0.05) and TLR2/6 ligand Pam_2_CSK_4_ stimulation (Fig 4b) resulted in upregulation of CXCL8 in BP- and HDM-allergic patients compared to the non-allergic donors (both p<0.05). Comparisons between non-stimulated samples of non-allergic and allergic donors were not significant. Stimulation with the TLR4 ligand LPS did not result in significant differences in CXCL8 secretion between the three groups, although single individuals displayed a strong CXCL8 response (Fig 4c).

### Activation marker expression and cyto-/chemokine secretion

The basophil activation marker CD203c was significantly upregulated upon stimulation with the two positive controls (anti-FcεRI antibody and fMLF) but not upon stimulation with TLR ligands (Fig 4d). A release of IL-6, IFN-γ and TNF-α was neither detected from unstimulated nor stimulated basophils with the exception of 1 to 3 donors from each group, which showed low secretion levels.

## Discussion

Innate immunity, especially TLR signaling, is increasingly considered crucial for influencing the development of allergic reactions[2, 3]. Hence, basophils, important cells of innate immunity and main elicitors of allergic symptoms, could be key cells in humans to link innate immune reactions to allergy[10, 15, 16]. In this study we investigated TLR expression of basophils and their stimulation-induced cyto-/chemokine secretion in seasonally affected BP-pollen- and perennially affected HDM-allergic patients compared to non-allergic donors. We observed enhanced CXCL8 secretion in BP-allergic patients despite of similar expression levels of TLR1 and 2 on basophils in allergic patients and non-allergic donors. In contrast, TLR4 was reduced in both allergic patient groups. This observation is consistent with a proposed role of TLR4 signaling in allergy development based on the findings of Hollingsworth *et al*.[16] who reported severe allergic inflammation in TLR4-deficient (C3H/HeJ) mice upon prolonged allergen stimulation. The fact that CXCL8 levels upon TLR stimulation of BP- and HDM-allergic patient groups were higher compared to those of non-allergic individuals adds to Gilmartin *et al*.[17] who additionally found increased CXCL8 expression upon FcεRI crosslinking in basophils of allergic donors, which they discussed as crucial for the recruitment of neutrophils, eosinophils, and monocytes to areas of inflammation. CXCL8 might, therefore, aggravate late-phase reactions by enhanced inflammatory cell recruitment to the sites of parasitic infection. According to our data, this could be mediated by the amplified CXCL8 secretion of basophils from the allergic patients upon TLR1/2 and TLR2/6 stimulation and, hence, boost the allergen-mediated inflammation as shown in the study of Gilmartin *et al*. Basophils were highly purified, however, a minor contribution in CXCL8 secretion by contaminating cells cannot be excluded.

As stimulation by TLR ligands is well-known to primarily induce secretion of pro-inflammatory cyto-/chemokines in a number of different cell types, we were interested in a potential induction of IFN-γ, TNF-α, CXCL8, and IL-6. With the exception of CXCL8, basophils did not respond with secretion of pro-inflammatory cyto-/chemokines. Our study stands in agreement with data recently described by Jeon *et al*.[18] who found IL-6 and CXCL8 secretion in flagellin-stimulated KU815 cells but not IFN-γ, IL-1β or TNF-α. Next to the selected cyto-/chemokine panel we analyzed the expression of CD203c, a very sensitive basophil activation marker, on the cell surface[19]. However, no effects of the TLR ligands on its expression could be detected, which indicates that basophils do not degranulate upon TLR stimulation.

In conclusion, elevated CXCL8 secretion upon TLR stimulation despite comparable TLR1 and 2 expression may represent an additional mechanism of how tissue inflammation might be exacerbated in allergic individuals and amplify allergic responses, like suggested by Yi *et al*.[20]
